# Value of Time to Positivity of Blood Culture in Children with Bloodstream Infections

**DOI:** 10.1155/2019/5975837

**Published:** 2019-01-10

**Authors:** Fen Pan, Wantong Zhao, Hong Zhang

**Affiliations:** Department of Clinical Laboratory, Shanghai Children's Hospital, Shanghai Jiaotong University, Shanghai, China

## Abstract

**Objective:**

This study was to investigate the microbiological characteristics and the relationship between the time to positivity (TTP) of blood cultures and different bacterial species and to assess the clinical value of TTP in children with bloodstream infections (BSIs).

**Methods:**

The TTP of all the blood cultures from children with suspected BSIs was retrospectively collected in 2016. The microbiological characteristics and the relationship between the TTP of blood cultures and different bacterial species were also analyzed.

**Results:**

A total of 808 strains were isolated from 15835 blood cultures collected, and 145 (17.9%) were Gram-negative, 636 (78.7%) were Gram-positive, and 27 (3.3%) were fungi. The bacteria were divided into definite pathogens (174), possible pathogens (592), fungi (27), and contaminants (15). The average TTP of all positive blood cultures was 30.97 and ranged from 3.23 h to 92.73 h. The TTP of Gram-negative strains was significantly shorter than that of Gram-positive strains (*P* < 0.001) and fungi (*P* = 0.032). The mean TTP for *E. coli* (15.60 h) was shortest within the group of Gram-negative isolates, and the mean TTP for *Streptococcus* (17.34 h) within the group of Gram-positive isolates. Significant difference of the TTP was detected in methicillin-resistant vs methicillin-susceptible *S. aureus*, extended-spectrum beta-lactamases (ESBLs) positive vs negative *Enterobacteriaceae*, and extensive drug-resistant and non-XDR *A. baumannii*. The median TTP in patients with BSI was significantly shorter than in those without it (*P* < 0.001). ROC curve analysis indicated that the TTP cutoff value of CoNS, *S. aureus*, *E. coli*, and *K. pneumoniae* was 22.72 h, 19.6 h, 18.58 h, and 16.43 h, respectively, with most sensitive and specific predictor of BSIs.

**Conclusions:**

Our data acknowledged that TTP is a valuable index for the early prognosis of BSIs. TTP not only provides additional utility as a general predictor of bacteria with smear result but also provides the implication of drug-resistant organisms.

## 1. Introduction

Bloodstream infections (BSIs) represent a major threat to the public health. On the basis of data reported, the annual incidence of BSIs was 189 per 100 000 person-years and the age-adjusted death rate has risen over the past decade [1, 2]. Blood culture, as the golden standard, is necessary for the diagnosis of BSIs, which can provide the information of pathogens including bacteria and fungi [3, 4]. As a parameter of blood culture, time to positivity (TTP), which is defined as the length of time from the beginning of culture incubation to the detection of bacterial growth by an automated system [5], has been proposed as a diagnostic and prognostic tool for BSIs.

The TTP is influenced by several factors including different bacterial species and initial bacterial concentration, which depends on the processing parameter. Under the similar inoculum, it is also well known that the speed of growth in culture media differs by the bacterial species [6]. Previous studies characterized the clinical value of TTP in *Enterobacteriaceae*, *Streptococcus pneumoniae*, *Staphylococcus* spp., and *Candida* spp. [7–11] and provided the early primary positive result to the clinicians. In addition, the early detection of organisms contributes to optimizing antimicrobial therapy for children [12, 13].

Therefore, the purpose of this study was to investigate the microbiological characteristics and the relationship between the TTP of blood cultures and different bacterial species, and to assess the clinical value of TTP in children with BSIs.

## 2. Materials and Methods

### 2.1. Study Design and Patients

A retrospective study was conducted at the clinical microbiology department in Shanghai Children's Hospital, one of the largest pediatric hospitals in Shanghai, China. Patients aged 1 to 14 years with suspected BSIs were enrolled between January 1st and December 31st in 2016. The clinical data including age, gender, department, clinical indexes, the species, and TTP of positive blood cultures were also recorded.

BSIs were defined according to the 2012 Surviving Sepsis Campaign guidelines [14, 15]. Patients were excluded for following reasons: incomplete information, patients less than 1 year old, samples less than 2 ml or more than 6 ml in volume, more than two species isolated from one-side bottle.

### 2.2. Culture Procedures

Blood collection was performed with two bottles from two sides, respectively, and conducted based on the Clinical and Laboratory Standard Institute (CLSI) H03-A6 document (presently replaced by the GP41-A6 document) [16]. A blood volume of 2–6 ml was collected by venepuncture, after the skin had been cleaned with 70% alcohol and inoculated into a BacT/Alert aerobic blood culture bottle. The bottle was then incubated immediately in a BACTEC FX200-automated blood culture system (BD Biosciences, America). The blood culture status either positive or negative on day 7 of incubation was considered as the final result.

The species of positive blood cultures were identified using the VITEK 2 Compact system (BioMérieux Inc., France). Definite pathogens, possible pathogens, and contaminants were defined as described previously [17]. Briefly, definite pathogens are organisms known to cause disease, such as *Streptococcus agalactiae*, *Staphylococcus aureus*, and Gram-negative organisms. Possible pathogens are organisms that can cause disease under special circumstances, such as coagulase-negative *Staphylococcus* (CoNS), *α*-haemolytic *Streptococcus*, and *γ*-haemolytic *Streptococcus*. Contaminants are organisms that rarely cause disease, such as *Corynebacteriu*m species and *Propionibacterium* species. Coagulase-negative *Staphylococcus* (CoNS) was divided into groups: one-side positivity (CoNS++) and two-side positivity (CoNS+). Antimicrobial susceptibility testing was performed by Kirby–Bauer disc diffusion, and the breakpoints used for interpretation were recommended by the Clinical and Laboratory Standards Institute (CLSI) 2017 [18].

### 2.3. Statistical Analysis

Statistical analysis was performed by using SPSS 19.0 for Windows (version 19.0; SPSS Inc., Chicago, IL, USA). The data were expressed as means ± standard deviations (SD) and were analyzed with Student's *t*-test. Categorical variables, expressed as numbers and percentages, were compared by the Chi-squared or Fisher's exact test. A value of *P* ≤ 0.05 was considered statistically significant. To test the prognostic value of TTPs to predict BSIs, the receiver operating characteristics (ROCs) were plotted using MedCalc 12.4.0 software.

## 3. Results

### 3.1. Characteristics of Microbiology

A total of 808 strains were isolated from 15835 blood cultures collected throughout the entire study period (Table 1). Of the 808 positive strains, 145 (17.9%) were Gram negative, 636 (78.7%) were Gram positive, and 27 (3.3%) were fungi. CoNS was mainly isolated, accounting for 61.9% (500/808). *K. pneumonia*e and *E. coli* were common in Gram-negative organisms. The mean age of these patients was 3.91 ± 3.40 years with most patients being male (474, 58.7%).

The bacteria were divided into definite pathogens (174), possible pathogens (592), fungi (27), and contaminants (15). *K. pneumonia*e (68), *S. aureu*s (29), and *E. coli* (26) were predominant organisms among definite pathogens. The CoNS was the predominant isolate in the possible pathogen group. After clinical assessment by clinicians, a majority of CoNS were considered as contaminants.

### 3.2. Relationship between TTP and Strains

The average TTP of all isolates was 30.97 h, with a range from 3.23 h to 92.73 h. The TTP of Gram-negative strains was significantly shorter than the TTP of Gram-positive strains (*P* < 0.001) and fungi (*P*=0.032). Among the group of Gram-negative isolates, the mean TTP for *E. coli* (15.60 h) was shortest, while the average TTP was observed in *Streptococcus* spp. (17.34 h) within the group of Gram-positive isolates. Meanwhile, significant difference for the mean TTP was detected between CoNS++ and CoNS+ groups (*P*=0.002).

The rate and distribution of each species throughout the 1, 2, and 3 days of culture are illustrated in Table 2. All microorganisms except for CoNS and fungi grew within the 2 days. The blood cultures with detected definite pathogens always became positive with the first 1 day, while the blood culture with detected possible pathogen was positive within 24 h–48 h.

### 3.3. Relationship between TTP and Common Resistant Phenotype

As shown in Table 3, the mean TTP for methicillin-resistant *Staphylococcus aureus* (MRSA) was 17.37 h versus 26.63 h for methicillin-susceptible *Staphylococcus aureus* (MSSA) (*P*=0.010). The average TTP of ESBL-positive *Enterobacteriaceae* was shorter than that of negative isolates (13.77 h vs. 19.98 h, *P*=0.004), which was also found between extensive drug-resistant (XDR) and non-XDR *A. baumanniii*. No significant difference was detected in MRCNS vs. MSCNS and PNSP vs. PSSP.

### 3.4. Clinical Value of TTP

The median TTP in patients with BSI was significantly shorter than the median in those without it (*P* < 0.001). ROC curve analysis indicated that the TTP cutoff value of all CoNS+, *S. aureu*s, *K. pneumonia*e, and *E. coli* was 22.72 h, 19.6 h, 18.58 h, and 16.43 h, respectively, with most sensitive and specific predictor of BSIs. The area under the ROC curve (AUC), sensitivity, specificity, positive predictive value (PPV), and negative predictive value (NPV) of TTP to identify BSIs were also calculated (Table 4).

## 4. Discussion

BSIs currently pose a significant cause of nosocomial infections and contribute substantially to morbidity and mortality worldwide [19, 20]. However, it is not easy to establish the diagnosis of BSIs since early clinical signs are mostly non-specific and inconclusive. Blood culture is considered as an important tool to the diagnosis and illness monitoring of BSIs, which can provide intuitionistic and accurate etiological index [21]. Although the identified pathogen is still the gold standard to diagnose BSIs, TTP may differentiate pathogens and contaminants in early.

In this retrospective study, we determined the TTP of 808 positive blood cultures from children. Mostly CoNS pathogens were isolated within 48 h. The shortest TTP was detected in *K. pneumoniae* (3.23 h) and longest TTP was in CoNS (92.73 h). As a previous study reported that few true BSIs were tested after 48 h of culture [22], our data found that mostly definite pathogens including *S. aureu*s, *S. pneumoniae*, *K. pneumoniae*, and *E. coli* were detected within 48 h and thus recommended as definite pathogen causing severe sepsis. However, about 60% of positive blood cultures yielded CNS, which is comparable with most other recent studies [23, 24]. The TTP of single and double positive for CoNS differed significantly (*P* < 0.05). Double positive for CoNS maybe the true pathogens that caused BSI, while mostly single CoNS was contaminant for indifference of blood culture by clinicians and inaccuracy blood sampling [25]. Despite other factors, the short TTP for blood culture can reminder us that it may be a true pathogen, and clinicians can make decision about the management of this patient.

Furthermore, we found another phenomenon that the TTPs were significantly different between patients with BSIs caused by drug-resistance pathogens and patients caused by drug-susceptible pathogens, especially in MRSA, ESBLs-positive *Enterobacteriaceae*, and XDR *A. baumannii* isolates. These results were similar to the findings of previous recent studies that used automated blood culture systems [7,26–28]. TTP could provide earlier detection of drug-resistant isolates, and it would also help clinicians to adjust the antibiotics based on characteristics of smear and TTP after the positivity alarm. Pardo et al. previously reported that few true BSIs were detected after 48 h of culture and thus recommended antibiotic de-escalation at 48 h [22].

In addition, the clinical value of TTP varied in different species. The TTP concerning to definite pathogens (*S. aureu*s, *E. coli*, and *K. pneumoniae*) were ≤19.6 h, ≤18.58 h, and ≤16.43 h, respectively, which were considered to be associated with best clinical prognosis. In general, one-side positive blood culture isolated with these strains is recommended as true pathogens that caused BSIs. However, on account of CoNS isolates, our study showed that the best cutoff of TTP was 22.72 h, which can distinguish the infections and containments. It was also similar to a report with TTP ≤23.6 h conducted by Lai et al. [29]. When isolated with CoNS with long TTPs, clinicians should combine other assistant examination including pro-calcitonin and clinical features.

## 5. Conclusions

Although the limitation of TTP always exists and the inoculum amount of blood from children can influence the TTP, our data acknowledged that TTP is a valuable index for early prognosis of BSIs. TTP not only provides additional utility as a general predictor of bacteria with smear result but also provides the implication of drug-resistant organisms, which is helpful to the adjustment of antibiotic therapy.

## Figures and Tables

**Table 1:** TTP of 808 blood culture positive strains.

Microorganisms	Number of strains	Proportion (%)	Average of TTP	Minimum TTP	Maximum TTP
Gram negative	145	17.9	17.61 ± 9.69	3.23	68.63
* K. pneumoniae*	68		16.29 ± 9.02	3.23	60.23
* E. coli*	26		15.60 ± 7.36	9.05	38.67
* P. aeruginosa*	12		20.40 ± 5.59	5.77	27.05
* A. baumannii*	11		19.45 ± 11.92	9.48	44.67
Other negative	28		20.93 ± 12.74	7.80	68.63

Gram positive	636	78.7	33.84 ± 14.19	4.28	92.73
CoNS++	87		33.91 ± 10.42	9.91	70.22
CoNS+	413		37.51 ± 14.04	13.68	92.73
* S. aureus*	29		23.44 ± 8.79	12.30	46.57
* Enterococcus*	53		23.54 ± 9.43	8.40	44.93
* Streptococcus*	39		17.34 ± 8.18	4.28	45.07
Other positive	15		30.63 ± 15.19	12.77	63.65

Fungi					
* Candida* spp.	27	3.3	35.68 ± 14.93	14.10	82.25

Total	808		30.97 ± 14.89	3.23	92.73

TTP: time to positivity; CoNS: coagulase-negative *Staphylococcus*.

**Table 2:** The rate and distribution of 808 positive strains in four time periods.

Microorganisms	Total	0–24 h		24–48 h		48–72 h		>72 h	
		No.	Proportion (%)	No.	Proportion (%)	No.	Proportion (%)	No.	Proportion (%)
CoNS+	413	61	14.8	263	63.7	78	18.9	11	2.7
CoNS++	87	13	14.9	65	74.7	9	10.3	0	0
*S. aureus*	29	18	62.1	11	37.9	0	0	0	0
*Enterococcus*	53	29	54.7	24	45.3	0	0	0	0
*Streptococcus*	39	33	84.6	6	15.4	0	0	0	0
Other positive	15	6	40	7	46.7	2	13.3	0	0
*K. pneumoniae*	68	61	89.7	6	8.8	1	1.5	0	0
*E. coli*	26	24	92.3	2	7.7	0	0	0	0
*P. aeruginosa*	12	11	91.7	1	8.3	0	0	0	0
*A. baumannii*	11	9	81.8	2	18.2	0	0	0	0
Other negative	28	20	71.4	7	25.0	1	3.6	0	0
Fungi	27	2	7.4	21	77.8	3	11.1	1	3.7
Total	808	287	35.5	415	51.4	94	11.6	12	1.5

CoNS: coagulase-negative *Staphylococcus*.

**Table 3:** Relationship between TTP and resistant phenotype.

Microorganisms	No.	Average of TTP	Minimum TTP	Maximum TTP	*t*	Value of *P*
CoNS						
MRCNS^*∗*^	402	36.55 ± 13.58	9.17	92.73	0.077	0.782
MSCNS^*∗*^	98	38.23 ± 13.31	14.07	86.35		

*S. aureus*						
MRSA^*∗*^	10	17.37 ± 4.25	12.3	35.1	7.671	0.010
MSSA^*∗*^	19	26.63 ± 8.94	14.1	46.57		

*S. pneumoniae*						
PNSP^*∗*^	3	17.91 ± 3.22	14.38	20.7	2.174	0.166
PSSP^*∗*^	11	19.35 ± 8.03	10.43	36.85		

*Enterobacteriaceae*						
ESBLs^*∗*^	59	13.77 ± 5.65	3.23	33.85	8.904	0.004
** **Non-ESBLs	36	19.98 ± 10.82	9.05	60.23		

*A. baumannii*						
XDR^*∗*^	5	13.09 ± 4.23	9.48	19.58	11.113	0.009
Non-XDR	6	24.74 ± 13.99	11.42	44.67		

TTP: time to positivity; CoNS: coagulase-negative *Staphylococcus;* MRSA: methicillin-resistant *Staphylococcus aureus*; MSSA: methicillin-susceptible *Staphylococcus aureus*; PNSP: penicillin-nonsusceptible *S. pneumoniae*; PSSP: penicillin-susceptible *S. pneumoniae*; ESBLs: extended spectrum beta-lactamases; XDR: extensive drug resistance; MRCNS: methicillin-resistant coagulase-negative *Staphylococcus;* MSCNS: methicillin-susceptible coagulase-negative *Staphylococcus*.^∗^Common resistant phenotypes in some bacteria in clinical process; the TTP of these bacteria could provide earlier detection of drug-resistant isolates, and it would also help clinicians to adjust the antibiotics based on characteristics of smear and TTP after the positivity alarm.

**Table 4:** Predictive value of TTP of microorganisms from bloodstream infections.

Microorganisms	AUC	Cutoff (h)	Sensitivity (%)	Specificity (%)	PPV (%)	NPV (%)
CoNS+	0.928	22.72	88.57	93.33	93	89.09
*S. aureus*	0.874	19.6	66.67	100	100	75
*K. pneumoniae*	0.818	18.58	76.67	75	75.41	71.19
*E. coli*	0.792	16.43	80	83.33	92.30	80.64

TTP: time to positivity; CoNS: coagulase-negative *Staphylococcus;* AUC: area under the ROC curve; PPV: positive predictive value; NPV: negative predictive value.

## Data Availability

The pdf data used to support the findings of this study are included in Supplementary Materials.

## Supplementary Materials

Supplementary Materials The supplementary materials include the TTPs of 880 positive blood culture, bacteria distribution, resistance phenotype of bacteria, and information of patients.

## References

[1] Uslan D. Z., Crane S. J., Steckelberg J. M. (2007). Age- and sex-associated trends in bloodstream infection. *Archives of Internal Medicine*.

[2] National Nosocomial Infections Surveillance (2000). National Nosocomial infections surveillance (NNIS) system report, data summary from January 1992-April 2000, issued June 2000. *American Journal of Infection Control*.

[3] Goto M., Al-Hasan M. N. (2013). Overall burden of bloodstream infection and nosocomial bloodstream infection in North America and Europe. *Clinical Microbiology and Infection*.

[4] Opota O., Jaton K., Greub G. (2015). Microbial diagnosis of bloodstream infection: towards molecular diagnosis directly from blood. *Clinical Microbiology and Infection*.

[5] Tang P. C., Lee C. C., Li C. W., Li M. C., Ko W. C., Lee N. Y. (2017). Time-to-positivity of blood culture: an independent prognostic factor of monomicrobial *Pseudomonas aeruginosa* bacteremia. *Journal of Microbiology, Immunology and Infection*.

[6] Defrance G., Birgand G., Ruppe E. (2013). Time-to-positivity-based discrimination between Enterobacteriaceae, *Pseudomonas aeruginosa* and strictly anaerobic Gram-negative bacilli in aerobic and anaerobic blood culture vials. *Journal of Microbiological Methods*.

[7] Alvarez R., Vinas-Castillo L., Lepe-Jimenez J. A., Garcia-Cabrera E., Cisneros-Herreros J. M. (2012). Time to positivity of blood culture association with clinical presentation, prognosis and ESBL-production in *Escherichia coli* bacteremia. *European Journal of Clinical Microbiology and Infectious Diseases*.

[8] Peralta G., Rodriguez-Lera M. J., Garrido J. C., Ansorena L., Roiz M. P. (2006). Time to positivity in blood cultures of adults with Streptococcus pneumoniae bacteremia. *BMC Infectious Diseases*.

[9] Lai C. C., Wang C. Y., Liu W. L., Hou C. C., Huang Y. T., Hsueh P. R. (2011). Time to blood culture positivity as a predictor of methicillin resistance in *Staphylococcus aureus* bacteremia. *Journal of Infection*.

[10] Nunes C. Z., Marra A. R., Edmond M. B., Da Silva Victor E. P. C., Pereira C. A. P. (2013). Time to blood culture positivity as a predictor of clinical outcome in patients with Candida albicans bloodstream infection. *BMC Infectious Diseases*.

[11] Al-Hasan M. N., Huskins W. C., Lahr B. D., Eckel-Passow J. E., Baddour L. M. (2010). Epidemiology and outcome of Gram-negative bloodstream infection in children: a population-based study. *Epidemiology and Infection*.

[12] Sabatier C., Garcia X., Ferrer R. (2015). Blood culture differential time to positivity enables safe catheter retention in suspected catheter-related bloodstream infection: a randomized controlled trial. *Medicina Intensiva*.

[13] Ning Y., Hu R., Yao G., Bo S. (2016). Time to positivity of blood culture and its prognostic value in bloodstream infection. *European Journal of Clinical Microbiology and Infectious Diseases*.

[14] Dellinger R. P., Levy M. M., Rhodes A. (2013). Surviving Sepsis Campaign: international guidelines for management of severe sepsis and septic shock. *Intensive Care Medicine*.

[15] Dien Bard J., Mcelvania Tekippe E. (2016). Diagnosis of bloodstream infections in children. *Journal of Clinical Microbiology*.

[16] CLSI (2007). CLSI H3-A6 document. *Procedures for the Collection of Diagnostic Blood Specimens by Venipuncture*.

[17] Garcia-Prats J. A., Cooper T. R., Schneider V. F., Stager C. E., Hansen T. N. (2000). Rapid detection of microorganisms in blood cultures of newborn infants utilizing an automated blood culture system. *Pediatrics*.

[18] CLSI (2017). *Performance Standards for Antimicrobial Susceptibility Testing; 27th Informational Supplement. 11. Clinical and Laboratory Standards Institute document M100-27*.

[19] Fram D., Taminato M., Ponzio V. (2014). Risk factors for morbidity and mortality of bloodstream infection in patients undergoing hemodialysis: a nested case-control study. *BMC Research Notes*.

[20] Olaechea P. M., Palomar M., Alvarez-Lerma F. (2013). Morbidity and mortality associated with primary and catheter-related bloodstream infections in critically ill patients. *Revista Espan˜ola de Quimioterapia*.

[21] Opota O., Croxatto A., Prod’hom G., Greub G. (2015). Blood culture-based diagnosis of bacteraemia: state of the art. *Clinical Microbiology and Infection*.

[22] Pardo J., Klinker K. P., Borgert S. J., Trikha G., Rand K. H., Ramphal R. (2014). Time to positivity of blood cultures supports antibiotic de-escalation at 48 hours. *Annals of Pharmacotherapy*.

[23] Oshitani Y., Ishikawa T., Murata K., Aoyagi Y., Yabe Y., Aoshima M. (2012). Clinical consideration of coagulase negative Staphylococci isolated in blood culture. *Kansenshogaku Zasshi*.

[24] Latif M., Usman J., Gilani M., Munir T., Mushtaq M., Anjum R. (2015). Coagulase negative staphylococci - a fast emerging threat. *Journal of Pakistan Medical Association*.

[25] Guerti K., Devos H., Ieven M. M., Mahieu L. M. (2011). Time to positivity of neonatal blood cultures: fast and furious?. *Journal of Medical Microbiology*.

[26] Ok H. S., Lee H. S., Park M. J. (2013). Predictors and clinical outcomes of persistent methicillin-resistant *Staphylococcus aureus* bacteremia: a prospective observational study. *Korean Journal of Internal Medicine*.

[27] Lai C. C., Wang C. Y., Liu W. L. (2011). Time to blood culture positivity as a predictor of drug resistance in Acinetobacter baumannii complex bacteremia. *Journal of Infection*.

[28] Huang L., Sun L., Yan Y. (2014). Time to positivity of blood culture is predictive for nosocomial infection and infectious endocarditis instead of other clinical characteristics and prognosis in Acintobacter baumannii bloodstream infection. *Journal of Infection*.

[29] Lai C. C., Wang C. Y., Liu W. L., Huang Y. T., Liao C. H., Hsueh P. R. (2011). Time to positivity in blood cultures of staphylococci: clinical significance in bacteremia. *Journal of Infection*.

