# Supplementary material for: Value of Time to Positivity of Blood Culture in Children with Bloodstream Infections
Source: Can J Infect Dis Med Microbiol. 2019 Jan 10;2019:5975837. doi: 10.1155/2019/5975837 (PMC6348829; doi:10.1155/2019/5975837)
Supplement: Supplementary Materials — The supplementary materials include the TTPs of 880 positive blood culture, bacteria distribution, resistance phenotype of bacteria, and information of patients. [file 5975837.f1.pdf]

NO.	Gender	Age(year)	Sample	Bacterial	Microorganisms	MM	BSI	TTP	Time period	ESBLS	CRE	MR	PRSP/PISP
1	m	3	bl	kpn	Klebsiella pneumoniae		1	16.85	1	+	+		
2			bl	sep	Staphylococcus epidermidis	CoNS++	0	46.58333333	2			MRCNS	
3			bl	sho	Staphylococcus hominis	CoNS+	0	18.16666667	1			MRCNS	
4			bl	sep	Staphylococcus epidermidis	CoNS+	0	86.35	4			MSCNS	
5			bl	sep	Staphylococcus epidermidis	CoNS+	0	60.26666667	3			MSCNS	
6			bl	sep	Staphylococcus epidermidis	CoNS+	0	47.78333333	2			MSCNS	
7			bl	sho	Staphylococcus hominis	CoNS+	0	46.68333333	2			MRCNS	
8			bl	sho	Staphylococcus hominis	CoNS+	0	22.25	1			MRCNS	
9	f	1	bl	pae	Pseudomonas aeruginosa		1	23.51666667	1				
10			bl	sho	Staphylococcus hominis	CoNS+	0	74.73333333	4			MRCNS	
11	m	5	bl	kpn	Klebsiella pneumoniae		1	41.03333333	2	-			
12			bl	sep	Staphylococcus epidermidis	CoNS+	0	48.91666667	3			MRCNS	
13			bl	sep	Staphylococcus epidermidis	CoNS+	0	71.7	3			MRCNS	
14			bl	shl	Staphylococcus haemolyticus	CoNS+	0	80.58333333	4			MRCNS	
15			bl	sep	Staphylococcus epidermidis	CoNS+	0	39.9	2			MSCNS	
16			bl	sho	Staphylococcus hominis	CoNS++	0	35	2			MSCNS	
17	m	1	bl	efa	Enterococcus faecalis		1	19.51666667	1				
18			bl	sep	Staphylococcus epidermidis	CoNS+	0	53.18333333	3			MRCNS	
19			bl	sho	Staphylococcus hominis	CoNS+	0	26.76666667	2			MRCNS	
20			bl	sho	Staphylococcus hominis	CoNS+	0	27.41666667	2			MRCNS	
21			bl	sho	Staphylococcus hominis	CoNS+	0	27.25	2			MRCNS	
22			bl	sho	Staphylococcus hominis	CoNS+	0	23.81666667	1			MRCNS	
23			bl	sep	Staphylococcus epidermidis	CoNS+	0	47.56666667	2			MRCNS	
24			bl	sep	Staphylococcus epidermidis	CoNS+	0	27.16666667	2			MRCNS	
25			bl	sho	Staphylococcus hominis	CoNS+	0	34.58333333	2			MRCNS	
26			bl	sep	Staphylococcus epidermidis	CoNS++	0	36.26666667	2			MRCNS	
27			bl	sho	Staphylococcus hominis	CoNS++	0	46.76666667	2			MRCNS	
28			bl	sep	Staphylococcus epidermidis	CoNS+	0	35.53333333	2			MRCNS	
29			bl	efa	Enterococcus faecalis		0	20.25	1				
30			bl	sep	Staphylococcus epidermidis	CoNS+	0	43.7	2			MRCNS	
31			bl	shl	Staphylococcus haemolyticus	CoNS+	0	50.3	3			MRCNS	
32			bl	sho	Staphylococcus hominis	CoNS+	0	44.41666667	2			MSCNS	
33			bl	sep	Staphylococcus epidermidis	CoNS+	0	45.4	2			MSCNS	
34	m	13	bl	efa	Enterococcus faecalis		1	16.46666667	1				
35			bl	sep	Staphylococcus epidermidis	CoNS+	0	34.68333333	2			MSCNS	
36			bl	shl	Staphylococcus haemolyticus	CoNS++	0	25.78333333	2			MRCNS	
37			bl	sho	Staphylococcus hominis	CoNS+	0	33.18333333	2			MSCNS	
38	m	4	bl	kpn	Klebsiella pneumoniae		1	22.88333334	1	-			

39			bl	sep	Staphylococcus epidermidis	CoNS+	0	35.45	2			MSCNS	
40			bl	sep	Staphylococcus epidermidis	CoNS+	0	40.48333333	2			MSCNS	
41			bl	sho	Staphylococcus hominis	CoNS+	0	23.3	1			MRCNS	
42	m	1	bl	egg	Enterobacter gergoviae		1	12.98333333	1				
43	m	1	bl	kpn	Klebsiella pneumoniae		1	17.91666667	1	+			
44			bl	sep	Staphylococcus epidermidis	CoNS++	0	34.56666667	2			MRCNS	
45	m	8	bl	eco	Escherichia coli		1	12.11666667	1	-			
46			bl	sep	Staphylococcus epidermidis	CoNS+	0	36.78333333	2			MRCNS	
47			bl	sep	Staphylococcus epidermidis	CoNS++	0	31.65	2			MRCNS	
48			bl	sho	Staphylococcus hominis	CoNS+	0	19.66666667	1			MRCNS	
49	m	13	bl	kpn	Klebsiella pneumoniae		1	12.98333333	1	+			
50			bl	sep	Staphylococcus epidermidis	CoNS+	0	39.98333333	2			MRCNS	
51	f	6	bl	shl	Staphylococcus haemolyticus	CoNS++	1	9.916666668	1			MRCNS	
52	f	2	bl	sho	Staphylococcus hominis	CoNS+	1	37.05	2			MRCNS	
53	m	2	bl	spn	Streptococcus Pneumoniae		1	18.65	1				PISP
54			bl	stc	Staphylococcus cohnii	CoNS++	0	46.03333333	2			MSCNS	
55	m	12	bl	kpn	Klebsiella pneumoniae		1	27.06666667	2	+			
56	f	1	bl	eco	Escherichia coli		1	13.31666667	1	+			
57			bl	sep	Staphylococcus epidermidis	CoNS+	0	33.8	2			MRCNS	
58			bl	sep	Staphylococcus epidermidis	CoNS+	0	47.61666667	2			MRCNS	
59			bl	shl	Staphylococcus haemolyticus	CoNS+	0	33.16666667	2			MRCNS	
60	m	1	bl	sep	Staphylococcus epidermidis	CoNS+	1	19.86666667	1			MRCNS	
61			bl	sep	Staphylococcus epidermidis	CoNS++	0	38.46666667	2			MRCNS	
62	f	7	bl	sep	Staphylococcus epidermidis	CoNS+	1	54.36666667	3			MRCNS	
63			bl	sho	Staphylococcus hominis	CoNS+	0	31.81666667	2			MRCNS	
64			bl	sho	Staphylococcus hominis	CoNS+	0	37.18333333	2			MRCNS	
65			bl	sep	Staphylococcus epidermidis	CoNS+	0	29.8	2			MRCNS	
66	m	4	bl	aba	Acinetobacter baumannii		1	44.66666667	2	NON-XDR			
67			bl	sep	Staphylococcus epidermidis	CoNS++	0	37.93333333	2			MSCNS	
68	f	13	bl	sep	Staphylococcus epidermidis	CoNS+	1	22.71666667	1			MRCNS	
69			bl	sep	Staphylococcus epidermidis	CoNS+	0	30.31666667	2			MRCNS	
70	m	3	bl	sep	Staphylococcus epidermidis	CoNS++	1	22.3	1			MRCNS	
71			bl	shl	Staphylococcus haemolyticus	CoNS+	0	17.36666667	1			MRCNS	
72	f	1	bl	efa	Enterococcus faecalis		1	14.73333333	1				
73	f	2	bl	eco	Escherichia coli		1	10.31666667	1	+			
74			bl	sep	Staphylococcus epidermidis	CoNS++	0	45.43333333	2			MRCNS	
75			bl	sau	Staphylococcus aureus		0	33.31666667	2			MSSA	
76			bl	sep	Staphylococcus epidermidis	CoNS+	0	32	2			MSCNS	
77			bl	efa	Enterococcus faecalis		0	20.8	1				
78			bl	ega	Enterococcus gallinarum		0	26.46666666	2				

79			bl	sep	Staphylococcus epidermidis	CoNS+	0	44.01666667	2			MSCNS	
80			bl	sho	Staphylococcus hominis	CoNS++	0	21.96666667	1			MRCNS	
81			bl	efm	Enterococcus faecium		0	32.5	2				
82			bl	ega	Enterococcus gallinarum		0	21.33333333	1				
83			bl	sep	Staphylococcus epidermidis	CoNS+	0	48.28333333	3			MRCNS	
84			bl	sep	Staphylococcus epidermidis	CoNS+	0	40.76666667	2			MRCNS	
85			bl	sho	Staphylococcus hominis	CoNS+	0	30.76666667	2			MRCNS	
86			bl	sep	Staphylococcus epidermidis	CoNS++	0	31.41666667	2			MRCNS	
87			bl	sho	Staphylococcus hominis	CoNS++	0	30.91666667	2			MRCNS	
88			bl	shl	Staphylococcus haemolyticus	CoNS+	0	27.83333333	2			MRCNS	
89			bl	sho	Staphylococcus hominis	CoNS+	0	45.11666667	2			MRCNS	
90	m	1	bl	kpn	Klebsiella pneumoniae		1	8.233333332	1	+			
91			bl	sho	Staphylococcus hominis	CoNS++	0	30.88333333	2			MRCNS	
92			bl	sho	Staphylococcus hominis	CoNS+	0	30.96666667	2			MSCNS	
93	m	6	bl	kpn	Klebsiella pneumoniae		1	17.2	1	+			
94			bl	sep	Staphylococcus epidermidis	CoNS+	0	20.95	1			MSCNS	
95			bl	sep	Staphylococcus epidermidis	CoNS+	0	29.13333333	2			MRCNS	
96			bl	sho	Staphylococcus hominis	CoNS+	0	32.86666667	2			MRCNS	
97			bl	sep	Staphylococcus epidermidis	CoNS+	0	32.7	2			MSCNS	
98			bl	sep	Staphylococcus epidermidis	CoNS+	0	41.63333334	2			MRCNS	
99			bl	shl	Staphylococcus haemolyticus	CoNS+	0	56	3			MRCNS	
100	m	4	bl	sau	Staphylococcus aureus		1	23.28333333	1			MSSA	
101			bl	sep	Staphylococcus epidermidis	CoNS+	0	51.86666667	3			MRCNS	
102			bl	sep	Staphylococcus epidermidis	CoNS+	0	26.33333333	2			MRCNS	
103	m	1	bl	eco	Escherichia coli		1	10.53333333	1	-			
104			bl	sep	Staphylococcus epidermidis	CoNS+	0	45.4	2			MSCNS	
105			bl	sho	Staphylococcus hominis	CoNS+	0	31.41666667	2			MRCNS	
106			bl	sep	Staphylococcus epidermidis	CoNS+	0	48.63333333	3			MRCNS	
107			bl	shl	Staphylococcus haemolyticus	CoNS+	0	36.48333333	2			MRCNS	
108			bl	efa	Enterococcus faecalis		0	23.15	1				
109			bl	sau	Staphylococcus aureus		0	23.15	1			MRSA	
110			bl	shl	Staphylococcus haemolyticus	CoNS+	0	20.05	1			MRCNS	
111			bl	sho	Staphylococcus hominis	CoNS+	0	52.95	3			MRCNS	
112			bl	sep	Staphylococcus epidermidis	CoNS+	0	42.35	2			MSCNS	
113			bl	sho	Staphylococcus hominis	CoNS+	0	16.13333333	1			MRCNS	
114			bl	sep	Staphylococcus epidermidis	CoNS+	0	26.7	2			MSCNS	
115			bl	sho	Staphylococcus hominis	CoNS+	0	27.3	2			MRCNS	
116			bl	sho	Staphylococcus hominis	CoNS+	0	21.85	1			MRCNS	
117			bl	efa	Enterococcus faecalis		0	17.95	1				
118			bl	sho	Staphylococcus hominis	CoNS+	0	32.4	2			MRCNS	

119			bl	sho	Staphylococcus hominis	CoNS+	0	22.65	1			MRCNS	
120	m	1	bl	kpn	Klebsiella pneumoniae		1	8.283333334	1	+			
121			bl	pan	Pantoea		0	25.3	2				
122	f	5	bl	efm	Enterococcus faecium		1	16.98333333	1				
123			bl	sep	Staphylococcus epidermidis	CoNS+	0	36.88333333	2			MRCNS	
124			bl	sho	Staphylococcus hominis	CoNS++	0	43.33333333	2			MRCNS	
125			bl	stc	Staphylococcus cohnii	CoNS++	0	38.88333333	2			MRCNS	
126			bl	sep	Staphylococcus epidermidis	CoNS+	0	23.2	1			MRCNS	
127			bl	sho	Staphylococcus hominis	CoNS+	0	46.06666667	2			MRCNS	
128			bl	sep	Staphylococcus epidermidis	CoNS+	0	26.3	2			MRCNS	
129			bl	sho	Staphylococcus hominis	CoNS+	0	25.68333333	2			MRCNS	
130			bl	sho	Staphylococcus hominis	CoNS+	0	25.56666667	2			MRCNS	
131	f	1	bl	sgc	Streptococcus agalactiae		1	16.43333333	1				
132			bl	sho	Staphylococcus hominis	CoNS+	0	38.26666667	2			MRCNS	
133			bl	sep	Staphylococcus epidermidis	CoNS+	0	30.83333333	2			MRCNS	
134	m	4	bl	shl	Staphylococcus haemolyticus	CoNS+	1	19.88333333	1			MRCNS	
135			bl	sit	Staphylococcus intermedius	CoNS+	0	27.21666667	2			MRCNS	
136			bl	sep	Staphylococcus epidermidis	CoNS+	0	32.33333333	2			MRCNS	
137	m	2	bl	sho	Staphylococcus hominis	CoNS++	1	26.71666667	2			MRCNS	
138			bl	sho	Staphylococcus hominis	CoNS++	0	39.3	2			MRCNS	
139			bl	shl	Staphylococcus haemolyticus	CoNS+	0	22.83333333	1			MRCNS	
140			bl	sho	Staphylococcus hominis	CoNS+	0	70.4	3			MRCNS	
141			bl	sep	Staphylococcus epidermidis	CoNS+	0	23.5	1			MRCNS	
142			bl	sep	Staphylococcus epidermidis	CoNS+	0	45.86666667	2			MRCNS	
143			bl	sho	Staphylococcus hominis	CoNS+	0	33.35	2			MSCNS	
144			bl	sho	Staphylococcus hominis	CoNS+	0	73.01666667	4			MRCNS	
145	m	9	bl	eco	Escherichia coli		1	20.9	1	+			
146			bl	sho	Staphylococcus hominis	CoNS+	0	34.71666667	2			MSCNS	
147			bl	sho	Staphylococcus hominis	CoNS++	0	37.51666667	2			MRCNS	
148			bl	sep	Staphylococcus epidermidis	CoNS+	0	41.05	2			MRCNS	
149			bl	sho	Staphylococcus hominis	CoNS+	0	28.16666667	2			MRCNS	
150			bl	sho	Staphylococcus hominis	CoNS+	0	32.16666666	2			MRCNS	
151	m	5	bl	sho	Staphylococcus hominis	CoNS+	1	19.18333333	1			MSCNS	
152	m	3	bl	spn	Streptococcus Pneumoniae		1	22.3	1				PSSP
153			bl	sho	Staphylococcus hominis	CoNS+	0	54.38333333	3			MRCNS	
154	m	6	bl	aba	Acinetobacter baumannii		1	18.03333333	1	NON-XDR			
155			bl	sep	Staphylococcus epidermidis	CoNS++	0	40.4	2			MRCNS	
156			bl	sho	Staphylococcus hominis	CoNS+	0	40.46666667	2			MRCNS	
157			bl	sep	Staphylococcus epidermidis	CoNS+	0	35.78333333	2			MRCNS	
158			bl	mlu	Micrococcus luteus		0	33.63333333	2				

159			bl	sho	Staphylococcus hominis	CoNS+	0	37.23333333	2			MRCNS	
160			bl	ssi	Staphylococcus simulans	CoNS+	0	27.45	2			MRCNS	
161	m	13	bl	efa	Enterococcus faecalis		1	15.98333333	1				
162			bl	sep	Staphylococcus epidermidis	CoNS+	0	62.3	3			MRCNS	
163			bl	sep	Staphylococcus epidermidis	CoNS+	0	31.46666667	2			MSCNS	
164			bl	sho	Staphylococcus hominis	CoNS+	0	52.93333333	3			MSCNS	
165			bl	sho	Staphylococcus hominis	CoNS+	0	29.13333333	2			MSCNS	
166			bl	sep	Staphylococcus epidermidis	CoNS++	0	39.18333333	2			MRCNS	
167			bl	shl	Staphylococcus haemolyticus	CoNS+	0	27.71666667	2			MRCNS	
168			bl	sho	Staphylococcus hominis	CoNS++	0	36.7	2			MRCNS	
169	m	1	bl	efa	Enterococcus faecalis		1	8.4	1				
170			bl	sho	Staphylococcus hominis	CoNS+	0	44.2	2			MSCNS	
171			bl	sho	Staphylococcus hominis	CoNS+	0	19.43333333	1			MRCNS	
172			bl	sep	Staphylococcus epidermidis	CoNS+	0	63.56666667	3			MSCNS	
173			bl	shl	Staphylococcus haemolyticus	CoNS+	0	28.66666667	2			MRCNS	
174			bl	sep	Staphylococcus epidermidis	CoNS+	0	59.5	3			MRCNS	
175			bl	sep	Staphylococcus epidermidis	CoNS+	0	32.16666667	2			MRCNS	
176	f	13	bl	efa	Enterococcus faecalis		1	37.35	2				
177	f	1	bl	efm	Enterococcus faecium		1	30.73333333	2				
178			bl	sep	Staphylococcus epidermidis	CoNS+	0	29.9	2			MRCNS	
179	f	4	bl	sgc	Streptococcus agalactiae		1	11.45	1				
180			bl	sho	Staphylococcus hominis	CoNS+	0	41.5	2			MSCNS	
181			bl	sho	Staphylococcus hominis	CoNS+	0	27.03333333	2			MSCNS	
182			bl	sho	Staphylococcus hominis	CoNS++	0	30.1	2			MRCNS	
183	m	2	bl	kpn	Klebsiella pneumoniae		1	12.71666667	1	-	+		
184			bl	sep	Staphylococcus epidermidis	CoNS+	0	34.08333333	2			MSCNS	
185			bl	shl	Staphylococcus haemolyticus	CoNS+	0	26.66666667	2			MRCNS	
186			bl	sho	Staphylococcus hominis	CoNS+	0	31.45	2			MRCNS	
187			bl	sep	Staphylococcus epidermidis	CoNS+	0	29.06666667	2			MSCNS	
188			bl	shl	Staphylococcus haemolyticus	CoNS+	0	45.85	2			MRCNS	
189			bl	sho	Staphylococcus hominis	CoNS+	0	24.86666667	2			MRCNS	
190	f	7	bl	sho	Staphylococcus hominis	CoNS+	1	19.35	1			MRCNS	
191			bl	sho	Staphylococcus hominis	CoNS+	0	38.53333333	2			MRCNS	
192	f	4	bl	aba	Acinetobacter baumannii		1	11.41666667	1	NON-XDR			
193	f	4	bl	efa	Enterococcus faecalis		1	11.41666667	1				
194			bl	sho	Staphylococcus hominis	CoNS+	0	21.51666667	1			MRCNS	
195	m	1	bl	aba	Acinetobacter baumannii		1	11.66666667	1	XDR			
196			bl	sau	Staphylococcus aureus		0	24.66666667	2			MSSA	
197			bl	sep	Staphylococcus epidermidis	CoNS++	0	30.36666667	2			MRCNS	
198			bl	sep	Staphylococcus epidermidis	CoNS+	0	31.75	2			MSCNS	

199			bl	sho	Staphylococcus hominis	CoNS++	0	45.06666666	2			MSCNS	
200			bl	sho	Staphylococcus hominis	CoNS+	0	32.15	2			MRCNS	
201			bl	sho	Staphylococcus hominis	CoNS+	0	22.41666667	1			MRCNS	
202			bl	sep	Staphylococcus epidermidis	CoNS+	0	34.55	2			MRCNS	
203			bl	shl	Staphylococcus haemolyticus	CoNS++	0	34.55	2			MRCNS	
204	m	5	bl	pma	Stenotrophomonas maltophilia		1	27.21666667	2				
205			bl	sep	Staphylococcus epidermidis	CoNS+	0	36.56666667	2			MRCNS	
206			bl	shl	Staphylococcus haemolyticus	CoNS+	0	24.26666667	2			MSCNS	
207			bl	sep	Staphylococcus epidermidis	CoNS+	0	41.11666667	2			MRCNS	
208			bl	sep	Staphylococcus epidermidis	CoNS+	0	44.23333333	2			MRCNS	
209			bl	scp	Staphylococcus caprae	CoNS+	0	38.58333333	2			MSCNS	
210			bl	sho	Staphylococcus hominis	CoNS+	0	28.43333334	2			MRCNS	
211	f	3	bl	shl	Staphylococcus haemolyticus	CoNS+	1	20.15	1			MRCNS	
212	f	7	bl	pae	Pseudomonas aeruginosa		1	22.41666666	1				
213			bl	sep	Staphylococcus epidermidis	CoNS+	0	37.03333333	2			MRCNS	
214			bl	shl	Staphylococcus haemolyticus	CoNS+	0	30.2	2			MRCNS	
215			bl	sep	Staphylococcus epidermidis	CoNS+	0	39.71666667	2			MSCNS	
216			bl	shl	Staphylococcus haemolyticus	CoNS+	0	37.36666667	2			MRCNS	
217			bl	sho	Staphylococcus hominis	CoNS++	0	30.75	2			MRCNS	
218			bl	sho	Staphylococcus hominis	CoNS+	0	56.9	3			MRCNS	
219			bl	efa	Enterococcus faecalis		0	36.61666667	2				
220	m	1	bl	kpn	Klebsiella pneumoniae		1	21.91666667	1	-	+		
221			bl	sep	Staphylococcus epidermidis	CoNS+	0	21.35	1			MRCNS	
222			bl	sep	Staphylococcus epidermidis	CoNS+	0	44.16666666	2			MRCNS	
223			bl	sho	Staphylococcus hominis	CoNS+	0	31.88333333	2			MRCNS	
224			bl	sep	Staphylococcus epidermidis	CoNS++	0	24.75	2			MRCNS	
225			bl	sho	Staphylococcus hominis	CoNS++	0	50.28333333	3			MRCNS	
226			bl	sho	Staphylococcus hominis	CoNS+	0	72.66666666	4			MSCNS	
227			bl	sep	Staphylococcus epidermidis	CoNS+	0	64.46666667	3			MRCNS	
228			bl	sep	Staphylococcus epidermidis	CoNS+	0	26.9	2			MRCNS	
229			bl	sep	Staphylococcus epidermidis	CoNS+	0	26.35	2			MRCNS	
230	m	1	bl	ecl	Enterobacter cloacae		1	20.15	1		+		
231			bl	efa	Enterococcus faecalis		0	10.25	1				
232			bl	sep	Staphylococcus epidermidis	CoNS++	0	19.63333333	1			MRCNS	
233			bl	sho	Staphylococcus hominis	CoNS+	0	37.75	2			MRCNS	
234			bl	sho	Staphylococcus hominis	CoNS+	0	27.5	2			MRCNS	
235			bl	efa	Enterococcus faecalis		0	13.13333333	1				
236			bl	sep	Staphylococcus epidermidis	CoNS+	0	51.38333333	3			MRCNS	
237			bl	sep	Staphylococcus epidermidis	CoNS+	0	25.73333333	2			MSCNS	
238			bl	sep	Staphylococcus epidermidis	CoNS+	0	36.66666667	2			MRCNS	

239	m	3	bl	sep	Staphylococcus epidermidis	CoNS+	1	13.68333333	1			MRCNS	
240			bl	sho	Staphylococcus hominis	CoNS+	0	30	2			MRCNS	
241			bl	sep	Staphylococcus epidermidis	CoNS+	0	70.18333333	3			MRCNS	
242			bl	sho	Staphylococcus hominis	CoNS+	0	58.45	3			MRCNS	
243			bl	sho	Staphylococcus hominis	CoNS+	0	75.01666667	4			MRCNS	
244			bl	shl	Staphylococcus haemolyticus	CoNS+	0	32.23333333	2			MRCNS	
245			bl	sho	Staphylococcus hominis	CoNS+	0	62.58333333	3			MRCNS	
246	f	1	bl	kox	Klebsiella oxytoca		1	18.31666667	1	-			
247	f	1	bl	kpn	Klebsiella pneumoniae		1	60.23333333	3	+			
248			bl	sho	Staphylococcus hominis	CoNS+	0	29.68333333	2			MSCNS	
249	m	8	bl	kpn	Klebsiella pneumoniae		1	16.1	1	-			
250	m	4	bl	kpn	Klebsiella pneumoniae		1	17.95	1	-			
251	f	6	bl	kpn	Klebsiella pneumoniae		1	12.01666667	1	-	+		
252			bl	sau	Staphylococcus aureus		0	30.9	2			MSSA	
253			bl	sep	Staphylococcus epidermidis	CoNS++	0	32.91666666	2			MRCNS	
254			bl	sep	Staphylococcus epidermidis	CoNS++	0	41.93333333	2			MSCNS	
255			bl	shl	Staphylococcus haemolyticus	CoNS+	0	24.41666667	2			MRCNS	
256			bl	sho	Staphylococcus hominis	CoNS++	0	32.75	2			MRCNS	
257			bl	sho	Staphylococcus hominis	CoNS++	0	28.61666667	2			MSCNS	
258			bl	sho	Staphylococcus hominis	CoNS+	0	44.88333333	2			MRCNS	
259			bl	sho	Staphylococcus hominis	CoNS+	0	29.3	2			MRCNS	
260			bl	sho	Staphylococcus hominis	CoNS+	0	27.95	2			MRCNS	
261	m	1	bl	kpn	Klebsiella pneumoniae		1	7.766666666	1	+	+		
262			bl	sep	Staphylococcus epidermidis	CoNS+	0	23.85	1			MRCNS	
263			bl	sho	Staphylococcus hominis	CoNS+	0	24.23333333	2			MRCNS	
264			bl	sho	Staphylococcus hominis	CoNS+	0	53.63333333	3			MRCNS	
265			bl	shl	Staphylococcus haemolyticus	CoNS+	0	38.66666667	2			MSCNS	
266	m	4	bl	eco	Escherichia coli		1	38.66666667	2	-			
267			bl	sau	Staphylococcus aureus		0	21.28333333	1			MSSA	
268			bl	sau	Staphylococcus aureus		0	35.1	2			MRSA	
269			bl	efa	Enterococcus faecalis		0	24.9	2				
270			bl	efa	Enterococcus faecalis		0	18.6	1				
271	m	1	bl	kpn	Klebsiella pneumoniae		1	18.6	1	-	+		
272			bl	sep	Staphylococcus epidermidis	CoNS+	0	42.51666667	2			MRCNS	
273	f	1	bl	eco	Escherichia coli		1	10.61666667	1	+			
274			bl	sho	Staphylococcus hominis	CoNS+	0	28.11666667	2			MRCNS	
275			bl	atu	Rhizobium radiobacter		0	29.21666667	2				
276	m	3	bl	sep	Staphylococcus epidermidis	CoNS+	1	20.91666667	1			MRCNS	
277			bl	sep	Staphylococcus epidermidis	CoNS+	0	30.06666667	2			MSCNS	
278			bl	sle	Staphylococcus sciuri	CoNS+	0	22.03333333	1			MRCNS	

279			bl	sep	Staphylococcus epidermidis	CoNS+	0	48.11666667	3			MRCNS	
280			bl	efm	Enterococcus faecium		0	35.58333333	2				
281			bl	pps	Staphylococcus saprophyticus	CoNS+	0	57.91666667	3			MRCNS	
282			bl	sep	Staphylococcus epidermidis	CoNS++	0	56.31666667	3			MRCNS	
283			bl	sho	Staphylococcus hominis	CoNS+	0	27.26666667	2			MRCNS	
284	f	1	bl	sho	Staphylococcus hominis	CoNS+	1	19.88333333	1			MRCNS	
285			bl	sho	Staphylococcus hominis	CoNS+	0	40.6	2			MRCNS	
286			bl	sho	Staphylococcus hominis	CoNS+	0	26.38333333	2			MRCNS	
287			bl	sep	Staphylococcus epidermidis	CoNS++	0	27.05	2			MRCNS	
288			bl	sho	Staphylococcus hominis	CoNS+	0	25.6	2			MRCNS	
289			bl	sho	Staphylococcus hominis	CoNS+	0	68.68333333	3			MSCNS	
290	m	4	bl	eco	Escherichia coli		1	33.85	2	+			
291			bl	sep	Staphylococcus epidermidis	CoNS+	0	80.06666667	4			MRCNS	
292			bl	shl	Staphylococcus haemolyticus	CoNS+	0	63.18333333	3			MRCNS	
293			bl	sep	Staphylococcus epidermidis	CoNS++	0	35.45	2			MRCNS	
294	f	5	bl	sgc	Streptococcus agalactiae		1	11.33333333	1				
295			bl	sho	Staphylococcus hominis	CoNS+	0	22.2	1			MRCNS	
296			bl	sho	Staphylococcus hominis	CoNS+	0	44.06666667	2			MRCNS	
297	m	5	bl	ssr	Staphylococcus sciuri	CoNS+	1	20.38333333	1			MRCNS	
298	f	3	bl	eco	Escherichia coli		1	9.433333334	1	-			
299			bl	sau	Staphylococcus aureus		0	20.38333333	1			MSSA	
300			bl	sep	Staphylococcus epidermidis	CoNS+	0	31.53333333	2			MRCNS	
301			bl	efm	Enterococcus faecium		0	26.15	2				
302	m	1	bl	kpn	Klebsiella pneumoniae		1	13.2	1	+			
303			bl	sep	Staphylococcus epidermidis	CoNS++	0	43.11666667	2			MRCNS	
304			bl	sep	Staphylococcus epidermidis	CoNS+	0	28.78333333	2			MRCNS	
305	f	1	bl	sep	Staphylococcus epidermidis	CoNS++	1	27.66666667	2			MRCNS	
306	f	1	bl	shl	Staphylococcus haemolyticus	CoNS++	1	14.31666667	1			MRCNS	
307	f	2	bl	kpn	Klebsiella pneumoniae		1	15.08333333	1	+			
308			bl	sep	Staphylococcus epidermidis	CoNS+	0	30.68333333	2			MRCNS	
309			bl	sep	Staphylococcus epidermidis	CoNS+	0	20.35	1			MSCNS	
310			bl	sep	Staphylococcus epidermidis	CoNS+	0	37.03333333	2			MRCNS	
311			bl	sep	Staphylococcus epidermidis	CoNS+	0	53.63333333	3			MRCNS	
312			bl	sep	Staphylococcus epidermidis	CoNS+	0	35.83333333	2			MRCNS	
313	f	1	bl	eco	Escherichia coli		1	9.250000001	1	+			
314			bl	sep	Staphylococcus epidermidis	CoNS+	0	31.25	2			MSCNS	
315			bl	sho	Staphylococcus hominis	CoNS+	0	31.45	2			MRCNS	
316			bl	sho	Staphylococcus hominis	CoNS+	0	43.18333333	2			MRCNS	
317			bl	swa	Staphylococcus warneri	CoNS+	0	39.63333333	2			MSCNS	
318	f	12	bl	eco	Escherichia coli		1	16.63333333	1	+			

319			bl	efm	Enterococcus faecium		0	24.1	2				
320	f	7	bl	kpn	Klebsiella pneumoniae		1	21.78333333	1	-			
321			bl	sho	Staphylococcus hominis	CoNS++	0	38.31666667	2			MRCNS	
322	m	1	bl	eco	Escherichia coli		1	22.76666667	1	-			
323			bl	sep	Staphylococcus epidermidis	CoNS+	0	34.16666667	2			MRCNS	
324	m	3	bl	aba	Acinetobacter baumannii		1	17.75	1	NON-XDR			
325	m	1	bl	kpn	Klebsiella pneumoniae		1	14.66666667	1	-			
326			bl	scp	Staphylococcus caprae	CoNS+	0	24.01666667	2			MSCNS	
327			bl	sep	Staphylococcus epidermidis	CoNS+	0	35.51666667	2			MRCNS	
328	f	1	bl	aba	Acinetobacter baumannii		1	9.483333331	1	XDR			
329	f	1	bl	efm	Enterococcus faecium		1	24.35	2				
330			bl	sep	Staphylococcus epidermidis	CoNS++	0	33.2	2			MRCNS	
331			bl	sep	Staphylococcus epidermidis	CoNS++	0	41.48333333	2			MRCNS	
332	f	4	bl	efa	Enterococcus faecalis		1	13.68333333	1				
333			bl	sep	Staphylococcus epidermidis	CoNS+	0	35.65	2			MRCNS	
334			bl	sep	Staphylococcus epidermidis	CoNS+	0	35.06666667	2			MRCNS	
335			bl	sho	Staphylococcus hominis	CoNS+	0	39.13333333	2			MRCNS	
336	f	3	bl	eco	Escherichia coli		1	17.06666667	1	+			
337	f	2	bl	sen	Salmonella		1	16.21666667	1				
338			bl	sep	Staphylococcus epidermidis	CoNS++	0	35.83333333	2			MRCNS	
339	m	1	bl	sgc	Streptococcus agalactiae		1	6.883333332	1				
340	f	1	bl	sau	Staphylococcus aureus		1	25.26666667	2			MRSA	
341			bl	sep	Staphylococcus epidermidis	CoNS+	0	54.36666667	3			MRCNS	
342	f	1	bl	kpn	Klebsiella pneumoniae		1	7.283333333	1	+			
343			bl	sep	Staphylococcus epidermidis	CoNS+	0	36.65	2			MRCNS	
344			bl	sep	Staphylococcus epidermidis	CoNS++	0	50.95	3			MSCNS	
345			bl	sho	Staphylococcus hominis	CoNS+	0	26.71666667	2			MSCNS	
346			bl	sho	Staphylococcus hominis	CoNS+	0	25.5	2			MRCNS	
347	m	2	bl	sho	Staphylococcus hominis	CoNS+	1	14.61666666	1			MRCNS	
348	f	2	bl	sgc	Streptococcus agalactiae		1	7.333333332	1				
349			bl	sho	Staphylococcus hominis	CoNS+	0	27.1	2			MRCNS	
350			bl	sho	Staphylococcus hominis	CoNS+	0	35.65	2			MRCNS	
351	f	1	bl	eco	Escherichia coli		1	9.683333335	1	+			
352			bl	sho	Staphylococcus hominis	CoNS+	0	35.45	2			MRCNS	
353			bl	sho	Staphylococcus hominis	CoNS+	0	47.63333333	2			MRCNS	
354			bl	efm	Enterococcus faecium		0	22.86666667	1				
355			bl	sca	Staphylococcus capitis	CoNS+	0	49.98333333	3			MRCNS	
356			bl	sep	Staphylococcus epidermidis	CoNS+	0	24.55	2			MRCNS	
357			bl	sho	Staphylococcus hominis	CoNS+	0	29.65	2			MRCNS	
358	f	1	bl	kpn	Klebsiella pneumoniae		1	11.3	1	+			

359	f	4	bl	sau	Staphylococcus aureus		1	14.1	1			MSSA	
360			bl	sep	Staphylococcus epidermidis	CoNS+	0	30.86666667	2			MSCNS	
361			bl	sho	Staphylococcus hominis	CoNS+	0	28.91666667	2			MRCNS	
362			bl	sho	Staphylococcus hominis	CoNS+	0	51.4	3			MRCNS	
363	f	1	bl	efa	Enterococcus faecalis		1	10.45	1				
364			bl	sep	Staphylococcus epidermidis	CoNS++	0	36.95	2			MRCNS	
365			bl	sho	Staphylococcus hominis	CoNS+	0	26.15	2			MRCNS	
366			bl	sho	Staphylococcus hominis	CoNS+	0	28.58333333	2			MRCNS	
367			bl	sep	Staphylococcus epidermidis	CoNS+	0	30.11666667	2			MRCNS	
368			bl	sep	Staphylococcus epidermidis	CoNS+	0	34.1	2			MRCNS	
369			bl	shl	Staphylococcus haemolyticus	CoNS+	0	43.3	2			MRCNS	
370			bl	sho	Staphylococcus hominis	CoNS+	0	24.31666667	2			MRCNS	
371			bl	efm	Enterococcus faecium		0	34.35	2				
372	m	1	bl	efm	Enterococcus faecium		1	27.05	2				
373			bl	sep	Staphylococcus epidermidis	CoNS+	0	53.03333334	3			MRCNS	
374			bl	sep	Staphylococcus epidermidis	CoNS+	0	53.01666667	3			MRCNS	
375	m	1	bl	sep	Staphylococcus epidermidis	CoNS+	1	15.2	1			MRCNS	
376	f	2	bl	kpn	Klebsiella pneumoniae		1	4.416666665	1	+	+		
377	m	1	bl	sep	Staphylococcus epidermidis	CoNS+	1	19.05	1			MRCNS	
378			bl	shl	Staphylococcus haemolyticus	CoNS+	0	56.45	3			MSCNS	
379			bl	sho	Staphylococcus hominis	CoNS+	0	26.7	2			MRCNS	
380	m	1	bl	sit	Staphylococcus intermedius	CoNS+	1	21.86666667	1			MSCNS	
381			bl	sep	Staphylococcus epidermidis	CoNS++	0	29.26666667	2			MRCNS	
382			bl	sep	Staphylococcus epidermidis	CoNS+	0	29.98333333	2			MRCNS	
383	f	1	bl	kpn	Klebsiella pneumoniae		1	3.233333335	1	+			
384	m	1	bl	kpn	Klebsiella pneumoniae		1	9.233333335	1	+			
385	f	1	bl	kpn	Klebsiella pneumoniae		1	10.98333333	1	+			
386			bl	sep	Staphylococcus epidermidis	CoNS+	0	36.71666667	2			MRCNS	
387			bl	sho	Staphylococcus hominis	CoNS+	0	52.05	3			MRCNS	
388	m	1	bl	kpn	Klebsiella pneumoniae		1	18.58333333	1	+			
389	f	1	bl	kpn	Klebsiella pneumoniae		1	18.6	1	+	+		
390	f	1	bl	kpn	Klebsiella pneumoniae		1	20.13333333	1	+			
391	m	1	bl	kpn	Klebsiella pneumoniae		1	21.75	1	+	+		
392			bl	pps	Staphylococcus saprophyticus	CoNS+	0	25.13333333	2			MRCNS	
393	m	2	bl	sep	Staphylococcus epidermidis	CoNS+	1	20.31666667	1			MRCNS	
394			bl	sep	Staphylococcus epidermidis	CoNS+	0	45.81666667	2			MSCNS	
395			bl	sep	Staphylococcus epidermidis	CoNS+	0	39.41666667	2			MRCNS	
396	m	8	bl	sgc	Streptococcus agalactiae		1	17.71666667	1				
397			bl	sho	Staphylococcus hominis	CoNS++	0	31.83333333	2			MRCNS	
398			bl	sep	Staphylococcus epidermidis	CoNS+	0	49.33333333	3			MRCNS	

399			bl	sep	Staphylococcus epidermidis	CoNS+	0	34.1	2			MRCNS	
400	m	1	bl	sgc	Streptococcus agalactiae		1	22.31666667	1				
401			bl	efm	Enterococcus faecium		0	40.2	2				
402			bl	sca	Staphylococcus capitis	CoNS+	0	16.55	1			MRCNS	
403			bl	sep	Staphylococcus epidermidis	CoNS+	0	34.66666667	2			MSCNS	
404			bl	sep	Staphylococcus epidermidis	CoNS++	0	41.61666666	2			MRCNS	
405			bl	shl	Staphylococcus haemolyticus	CoNS+	0	64	3			MRCNS	
406			bl	sca	Staphylococcus capitis	CoNS+	0	60.83333333	3			MSCNS	
407	m	2	bl	sal	Salmonella		1	36.65	2				
408			bl	sep	Staphylococcus epidermidis	CoNS++	0	49.13333333	3			MRCNS	
409			bl	sho	Staphylococcus hominis	CoNS++	0	27.78333333	2			MRCNS	
410			bl	sho	Staphylococcus hominis	CoNS+	0	44.18333333	2			MRCNS	
411			bl	sep	Staphylococcus epidermidis	CoNS++	0	36.36666667	2			MRCNS	
412			bl	sle	Staphylococcus sciuri	CoNS++	0	36.46666667	2			MRCNS	
413	f	2	bl	efa	Enterococcus faecalis		1	23.98333333	1				
414			bl	sho	Staphylococcus hominis	CoNS+	0	57.15	3			MRCNS	
415	f	10	bl	sau	Staphylococcus aureus		1	20.4	1			MSSA	
416			bl	sep	Staphylococcus epidermidis	CoNS+	0	50.11666667	3			MRCNS	
417	f	2	bl	sep	Staphylococcus epidermidis	CoNS+	1	21.35	1			MRCNS	
418			bl	sep	Staphylococcus epidermidis	CoNS+	0	42.45	2			MRCNS	
419			bl	sho	Staphylococcus hominis	CoNS+	0	26.96666667	2			MRCNS	
420			bl	sho	Staphylococcus hominis	CoNS+	0	45.26666667	2			MSCNS	
421			bl	sep	Staphylococcus epidermidis	CoNS+	0	34.15	2			MRCNS	
422			bl	shl	Staphylococcus haemolyticus	CoNS+	0	38.31666666	2			MSCNS	
423			bl	sep	Staphylococcus epidermidis	CoNS+	0	43.36666667	2			MRCNS	
424			bl	shl	Staphylococcus haemolyticus	CoNS+	0	27.5	2			MRCNS	
425			bl	sho	Staphylococcus hominis	CoNS+	0	40.58333333	2			MRCNS	
426			bl	sep	Staphylococcus epidermidis	CoNS+	0	43.86666667	2			MRCNS	
427			bl	sep	Staphylococcus epidermidis	CoNS+	0	36.45	2			MRCNS	
428			bl	sep	Staphylococcus epidermidis	CoNS+	0	26.13333333	2			MRCNS	
429			bl	sho	Staphylococcus hominis	CoNS++	0	20.36666667	1			MRCNS	
430	f	1	bl	pae	Pseudomonas aeruginosa		1	23.6	1		+		
431	f	1	bl	sgc	Streptococcus agalactiae		1	45.06666667	2				
432			bl	sep	Staphylococcus epidermidis	CoNS+	0	36.01666667	2			MRCNS	
433			bl	sho	Staphylococcus hominis	CoNS+	0	23.2	1			MRCNS	
434			bl	shl	Staphylococcus haemolyticus	CoNS+	0	37.48333333	2			MRCNS	
435			bl	sep	Staphylococcus epidermidis	CoNS+	0	53.71666667	3			MRCNS	
436			bl	shl	Staphylococcus haemolyticus	CoNS+	0	50.13333333	3			MRCNS	
437			bl	sep	Staphylococcus epidermidis	CoNS+	0	29.41666667	2			MRCNS	
438			bl	ssi	Staphylococcus simulans	CoNS+	0	30.25	2			MRCNS	

439			bl	shl	Staphylococcus haemolyticus	CoNS+	0	35.15	2			MRCNS	
440	f	6	bl	sgc	Streptococcus agalactiae		1	15.68333334	1				
441			bl	sho	Staphylococcus hominis	CoNS+	0	41.38333333	2			MRCNS	
442	m	2	bl	pae	Pseudomonas aeruginosa		1	19.21666667	1				
443	m	10	bl	sau	Staphylococcus aureus		1	16.5	1			MSSA	
444			bl	shl	Staphylococcus haemolyticus	CoNS+	0	51.68333334	3			MRCNS	
445	m	2	bl	sho	Staphylococcus hominis	CoNS+	1	21.48333333	1			MRCNS	
446			bl	sho	Staphylococcus hominis	CoNS+	0	25.55	2			MRCNS	
447			bl	sho	Staphylococcus hominis	CoNS+	0	33.26666667	2			MRCNS	
448			bl	sep	Staphylococcus epidermidis	CoNS++	0	40.1	2			MRCNS	
449			bl	sau	Staphylococcus aureus		0	46.56666666	2			MSSA	
450			bl	sep	Staphylococcus epidermidis	CoNS+	0	49.11666667	3			MRCNS	
451			bl	sho	Staphylococcus hominis	CoNS+	0	33.5	2			MRCNS	
452	f	2	bl	pma	Stenotrophomonas maltophilia		1	45.01666667	2				
453			bl	sep	Staphylococcus epidermidis	CoNS+	0	44.76666666	2			MRCNS	
454	f	1	bl	sau	Staphylococcus aureus		1	19.6	1			MRSA	
455			bl	shl	Staphylococcus haemolyticus	CoNS+	0	28.61666667	2			MRCNS	
456			bl	sep	Staphylococcus epidermidis	CoNS+	0	68.43333333	3			MRCNS	
457	m	3	bl	sho	Staphylococcus hominis	CoNS+	1	18.51666667	1			MRCNS	
458	f	1	bl	sho	Staphylococcus hominis	CoNS+	1	18.61666667	1			MRCNS	
459	f	3	bl	kpn	Klebsiella pneumoniae		1	12.05	1	-			
460	f	5	bl	pae	Pseudomonas aeruginosa		1	17.83333333	1				
461			bl	sep	Staphylococcus epidermidis	CoNS+	0	24.43333333	2			MRCNS	
462	f	1	bl	eco	Escherichia coli		1	16.36666667	1	-			
463			bl	sep	Staphylococcus epidermidis	CoNS+	0	56.95	3			MRCNS	
464	f	2	bl	spn	Streptococcus Pneumoniae		1	14.38333333	1				PISP
465	m	9	bl	sau	Staphylococcus aureus		1	16.15	1			MSSA	
466			bl	sho	Staphylococcus hominis	CoNS+	0	46.35	2			MRCNS	
467	f	1	bl	eco	Escherichia coli		1	9.050000002	1	-			
468			bl	sho	Staphylococcus hominis	CoNS++	0	41.01666666	2			MRCNS	
469	m	2	bl	efa	Enterococcus faecalis		1	16.11666666	1				
470			bl	sep	Staphylococcus epidermidis	CoNS++	0	20.63333333	1			MRCNS	
471	m	1	bl	efa	Enterococcus faecalis		1	23.36666667	1				
472			bl	sep	Staphylococcus epidermidis	CoNS+	0	23.65	1			MRCNS	
473			bl	sep	Staphylococcus epidermidis	CoNS++	0	14.06666667	1			MSCNS	
474	f	2	bl	sgc	Streptococcus agalactiae		1	13.21666667	1				
475			bl	sep	Staphylococcus epidermidis	CoNS+	0	51.55	3			MRCNS	
476			bl	sep	Staphylococcus epidermidis	CoNS+	0	27.86666667	2			MRCNS	
477			bl	efm	Enterococcus faecium		0	44.65	2				
478	m	1	bl	pae	Pseudomonas aeruginosa		1	22.43333333	1				

479			bl	sep	Staphylococcus epidermidis	CoNS+	0	40.21666667	2			MRCNS	
480			bl	sep	Staphylococcus epidermidis	CoNS+	0	40.53333333	2			MRCNS	
481			bl	sep	Staphylococcus epidermidis	CoNS+	0	59.16666667	3			MRCNS	
482			bl	sho	Staphylococcus hominis	CoNS+	0	36.9	2			MRCNS	
483			bl	sho	Staphylococcus hominis	CoNS+	0	26.3	2			MRCNS	
484	m	1	bl	pae	Pseudomonas aeruginosa		1	23.3	1				
485			bl	sep	Staphylococcus epidermidis	CoNS++	0	18.1	1			MRCNS	
486			bl	sep	Staphylococcus epidermidis	CoNS++	0	56.41666667	3			MRCNS	
487			bl	sep	Staphylococcus epidermidis	CoNS+	0	30.91666667	2			MSCNS	
488	m	8	bl	kpn	Klebsiella pneumoniae		1	12.96666667	1	+	+		
489			bl	scp	Staphylococcus caprae	CoNS+	0	51.03333333	3			MSCNS	
490			bl	sep	Staphylococcus epidermidis	CoNS+	0	57.23333333	3			MRCNS	
491	m	1	bl	eco	Escherichia coli		1	21.63333333	1	-			
492			bl	sep	Staphylococcus epidermidis	CoNS+	0	52.81666667	3			MRCNS	
493			bl	shl	Staphylococcus haemolyticus	CoNS+	0	44.58333333	2			MRCNS	
494			bl	sho	Staphylococcus hominis	CoNS+	0	43.65	2			MRCNS	
495			bl	sho	Staphylococcus hominis	CoNS+	0	30.33333333	2			MRCNS	
496			bl	pps	Staphylococcus saprophyticus	CoNS+	0	23.93333333	1			MRCNS	
497			bl	sho	Staphylococcus hominis	CoNS+	0	40.9	2			MSCNS	
498			bl	sho	Staphylococcus hominis	CoNS+	0	48.18333333	3			MSCNS	
499	f	1	bl	kpn	Klebsiella pneumoniae		1	17.4	1	+			
500			bl	mlu	Micrococcus luteus		0	44.45	2				
501			bl	sep	Staphylococcus epidermidis	CoNS++	0	22.56666667	1			MRCNS	
502			bl	sho	Staphylococcus hominis	CoNS++	0	27.06666667	2			MSCNS	
503			bl	sho	Staphylococcus hominis	CoNS+	0	22.1	1			MSCNS	
504			bl	sho	Staphylococcus hominis	CoNS+	0	22.43333333	1			MRCNS	
505	f	2	bl	kpn	Klebsiella pneumoniae		1	23.71666667	1	+	+		
506	f	8	bl	pae	Pseudomonas aeruginosa		1	15.06666667	1				
507			bl	sho	Staphylococcus hominis	CoNS+	0	33.38333333	2			MRCNS	
508			bl	sho	Staphylococcus hominis	CoNS+	0	33.7	2			MRCNS	
509	m	3	bl	sma	Serratia marcescens		1	7.8	1				
510			bl	sep	Staphylococcus epidermidis	CoNS+	0	36.86666667	2			MRCNS	
511	f	2	bl	kpn	Klebsiella pneumoniae		1	10.66666667	1	+			
512			bl	pps	Staphylococcus saprophyticus	CoNS++	0	49.28333333	3			MRCNS	
513	m	9	bl	sal	Salmonella		1	14.08333333	1				
514			bl	sep	Staphylococcus epidermidis	CoNS+	0	47.1	2			MRCNS	
515	f	1	bl	sgc	Streptococcus agalactiae		1	25.08333333	2				
516	f	2	bl	sho	Staphylococcus hominis	CoNS+	1	21.85	1			MSCNS	
517	f	2	bl	kpn	Klebsiella pneumoniae		1	7.266666665	1	+			
518			bl	sep	Staphylococcus epidermidis	CoNS+	0	62.71666667	3			MRCNS	

519	f	2	bl	sep	Staphylococcus epidermidis	CoNS+	1	18.6	1			MRCNS	
520			bl	sle	Staphylococcus sciuri	CoNS+	0	30.73333333	2			MRCNS	
521			bl	sep	Staphylococcus epidermidis	CoNS+	0	30.83333333	2			MRCNS	
522			bl	sho	Staphylococcus hominis	CoNS+	0	36.51666666	2			MSCNS	
523			bl	sep	Staphylococcus epidermidis	CoNS+	0	53.93333333	3			MRCNS	
524			bl	sho	Staphylococcus hominis	CoNS+	0	46.96666667	2			MSCNS	
525			bl	sho	Staphylococcus hominis	CoNS+	0	29.25	2			MSCNS	
526			bl	sho	Staphylococcus hominis	CoNS+	0	34.43333333	2			MSCNS	
527			bl	sca	Staphylococcus capitis	CoNS+	0	53.4	3			MRCNS	
528			bl	shl	Staphylococcus haemolyticus	CoNS+	0	20.43333333	1			MSCNS	
529			bl	sca	Staphylococcus capitis	CoNS++	0	42.03333333	2			MRCNS	
530			bl	shl	Staphylococcus haemolyticus	CoNS+	0	26.01666667	2			MRCNS	
531			bl	sho	Staphylococcus hominis	CoNS++	0	30.2	2			MSCNS	
532			bl	sho	Staphylococcus hominis	CoNS+	0	57.23333333	3			MSCNS	
533			bl	sho	Staphylococcus hominis	CoNS+	0	22.7	1			MRCNS	
534			bl	sep	Staphylococcus epidermidis	CoNS+	0	48.46666667	3			MRCNS	
535	f	1	bl	kpn	Klebsiella pneumoniae		1	10.51666667	1	+	+		
536	f	7	bl	sau	Staphylococcus aureus		1	15.9	1			MRSA	
537			bl	sep	Staphylococcus epidermidis	CoNS+	0	17.53333333	1			MRCNS	
538			bl	sep	Staphylococcus epidermidis	CoNS++	0	35.78333334	2			MRCNS	
539			bl	shl	Staphylococcus haemolyticus	CoNS++	0	32.3	2			MRCNS	
540			bl	sho	Staphylococcus hominis	CoNS+	0	23.8	1			MRCNS	
541			bl	ssi	Staphylococcus simulans	CoNS++	0	21.25	1			MSCNS	
542	f	1	bl	eco	Escherichia coli		1	18.03333333	1	+			
543			bl	pps	Staphylococcus saprophyticus	CoNS+	0	24.55	2			MRCNS	
544			bl	sca	Staphylococcus capitis	CoNS+	0	41.28333333	2			MSCNS	
545			bl	sep	Staphylococcus epidermidis	CoNS+	0	35.5	2			MRCNS	
546			bl	sep	Staphylococcus epidermidis	CoNS+	0	40.08333333	2			MRCNS	
547	f	1	bl	sgc	Streptococcus agalactiae		1	14.93333333	1				
548	f	4	bl	scp	Staphylococcus caprae	CoNS+	1	16.98333333	1			MSCNS	
549			bl	sep	Staphylococcus epidermidis	CoNS+	0	33.01666667	2			MSCNS	
550			bl	sho	Staphylococcus hominis	CoNS+	0	49.13333333	3			MRCNS	
551	f	1	bl	efm	Enterococcus faecium		1	25.88333333	2				
552			bl	sep	Staphylococcus epidermidis	CoNS+	0	92.73333333	4			MRCNS	
553			bl	sho	Staphylococcus hominis	CoNS+	0	31.73333334	2			MRCNS	
554			bl	sho	Staphylococcus hominis	CoNS+	0	40.56666667	2			MRCNS	
555	m	3	bl	sma	Serratia marcescens		1	13.41666667	1				
556			bl	sca	Staphylococcus capitis	CoNS+	0	65.28333333	3			MRCNS	
557			bl	sep	Staphylococcus epidermidis	CoNS++	0	31.25	2			MRCNS	
558			bl	sho	Staphylococcus hominis	CoNS+	0	32.18333333	2			MRCNS	

559	m	2	bl	sal	Salmonella		1	9.733333335	1				
560			bl	sep	Staphylococcus epidermidis	CoNS+	0	34.91666667	2			MRCNS	
561	m	2	bl	aba	Acinetobacter baumannii		1	14.93333333	1	XDR			
562			bl	sep	Staphylococcus epidermidis	CoNS+	0	34.45	2			MRCNS	
563			bl	shl	Staphylococcus haemolyticus	CoNS+	0	29.01666667	2			MRCNS	
564			bl	sho	Staphylococcus hominis	CoNS+	0	36.06666667	2			MSCNS	
565			bl	sep	Staphylococcus epidermidis	CoNS+	0	52.6	3			MRCNS	
566	m	1	bl	sgc	Streptococcus agalactiae		1	11.93333333	1				
567			bl	sep	Staphylococcus epidermidis	CoNS+	0	29.48333333	2			MRCNS	
568	m	4	bl	sho	Staphylococcus hominis	CoNS+	1	21.95	1			MRCNS	
569			bl	efm	Enterococcus faecium		0	34.46666667	2				
570			bl	sep	Staphylococcus epidermidis	CoNS+	0	33.95	2			MRCNS	
571			bl	sho	Staphylococcus hominis	CoNS+	0	26.4	2			MSCNS	
572	m	1	bl	efa	Enterococcus faecalis		1	17.71666667	1				
573	m	2	bl	kpn	Klebsiella pneumoniae		1	31.45	2	+			
574			bl	sep	Staphylococcus epidermidis	CoNS+	0	30.01666667	2			MRCNS	
575			bl	sho	Staphylococcus hominis	CoNS++	0	52.63333333	3			MRCNS	
576			bl	shl	Staphylococcus haemolyticus	CoNS+	0	48.93333333	3			MRCNS	
577	m	8	bl	spn	Streptococcus Pneumoniae		1	22.2	1				PSSP
578	m	1	bl	shl	Staphylococcus haemolyticus	CoNS+	1	17.18333334	1			MRCNS	
579			bl	ssi	Staphylococcus simulans	CoNS+	0	27.55	2			MRCNS	
580			bl	swa	Staphylococcus warneri	CoNS+	0	30.6	2			MRCNS	
581	m	2	bl	kpn	Klebsiella pneumoniae		1	23.18333333	1	-			
582			bl	sep	Staphylococcus epidermidis	CoNS+	0	41.65	2			MRCNS	
583			bl	sho	Staphylococcus hominis	CoNS+	0	24.21666667	2			MRCNS	
584			bl	sho	Staphylococcus hominis	CoNS+	0	28.46666667	2			MRCNS	
585	f	2	bl	kpn	Klebsiella pneumoniae		1	15.66666667	1	+			
586			bl	sep	Staphylococcus epidermidis	CoNS++	0	24.58333333	2			MRCNS	
587			bl	shl	Staphylococcus haemolyticus	CoNS++	0	24.98333333	2			MRCNS	
588	m	1	bl	spn	Streptococcus Pneumoniae		1	15.71666667	1				PSSP
589			bl	ssi	Staphylococcus simulans	CoNS++	0	30.15	2			MRCNS	
590	m	1	bl	eco	Escherichia coli		1	11.43333333	1	-			
591	f	2	bl	efa	Enterococcus faecalis		1	10.71666667	1				
592			bl	sep	Staphylococcus epidermidis	CoNS+	0	59.6	3			MSCNS	
593			bl	sep	Staphylococcus epidermidis	CoNS+	0	62.96666667	3			MSCNS	
594	m	2	bl	sho	Staphylococcus hominis	CoNS+	1	16.35	1			MRCNS	
595	m	2	bl	kpn	Klebsiella pneumoniae		1	8.966666666	1	+			
596			bl	sep	Staphylococcus epidermidis	CoNS+	0	60.13333333	3			MRCNS	
597	f	2	bl	sho	Staphylococcus hominis	CoNS+	1	16.15	1			MRCNS	
598			bl	sho	Staphylococcus hominis	CoNS+	0	57	3			MRCNS	

599	f	2	bl	kpn	Klebsiella pneumoniae		1	10.13333333	1	+			
600	f	3	bl	spn	Streptococcus Pneumoniae		1	21.63333333	1				PSSP
601			bl	sca	Staphylococcus capitis	CoNS+	0	77.51666667	4			MRCNS	
602			bl	sho	Staphylococcus hominis	CoNS+	0	35.81666667	2			MRCNS	
603	f	2	bl	kpn	Klebsiella pneumoniae		1	10.26666667	1	+	+		
604			bl	sep	Staphylococcus epidermidis	CoNS+	0	58.66666667	3			MRCNS	
605			bl	sep	Staphylococcus epidermidis	CoNS+	0	27.03333333	2			MRCNS	
606			bl	sep	Staphylococcus epidermidis	CoNS+	0	44.11666667	2			MSCNS	
607			bl	shl	Staphylococcus haemolyticus	CoNS+	0	31.41666667	2			MRCNS	
608			bl	sho	Staphylococcus hominis	CoNS+	0	32.81666667	2			MSCNS	
609			bl	swa	Staphylococcus warneri	CoNS+	0	40	2			MRCNS	
610			bl	sep	Staphylococcus epidermidis	CoNS+	0	57.38333334	3			MSCNS	
611			bl	sep	Staphylococcus epidermidis	CoNS++	0	70.21666667	3			MSCNS	
612			bl	sep	Staphylococcus epidermidis	CoNS+	0	41.1	2			MRCNS	
613			bl	sep	Staphylococcus epidermidis	CoNS+	0	49.01666667	3			MRCNS	
614			bl	shl	Staphylococcus haemolyticus	CoNS+	0	33.33333333	2			MSCNS	
615			bl	sho	Staphylococcus hominis	CoNS+	0	50.48333333	3			MRCNS	
616	m	14	bl	efa	Enterococcus faecalis		1	31.56666666	2				
617			bl	sep	Staphylococcus epidermidis	CoNS++	0	48.23333333	3			MRCNS	
618			bl	shl	Staphylococcus haemolyticus	CoNS++	0	32.86666667	2			MRCNS	
619			bl	sho	Staphylococcus hominis	CoNS+	0	31.76666667	2			MRCNS	
620	m	1	bl	kpn	Klebsiella pneumoniae		1	19.26666667	1	+	+		
621	m	12	bl	sau	Staphylococcus aureus		1	14.2	1			MRSA	
622			bl	sep	Staphylococcus epidermidis	CoNS++	0	28.51666667	2			MRCNS	
623			bl	shl	Staphylococcus haemolyticus	CoNS++	0	25.51666667	2			MRCNS	
624			bl	sho	Staphylococcus hominis	CoNS+	0	30.08333333	2			MRCNS	
625			bl	sep	Staphylococcus epidermidis	CoNS+	0	28.15	2			MRCNS	
626			bl	sho	Staphylococcus hominis	CoNS+	0	36.4	2			MSCNS	
627	f	1	bl	sgc	Streptococcus agalactiae		1	29.98333333	2				
628			bl	shl	Staphylococcus haemolyticus	CoNS+	0	34.81666667	2			MRCNS	
629			bl	sho	Staphylococcus hominis	CoNS+	0	25.73333334	2			MRCNS	
630			bl	sho	Staphylococcus hominis	CoNS+	0	25.26666667	2			MRCNS	
631			bl	sle	Staphylococcus sciuri	CoNS+	0	32.43333333	2			MRCNS	
632			bl	sho	Staphylococcus hominis	CoNS+	0	25.56666667	2			MRCNS	
633			bl	sho	Staphylococcus hominis	CoNS+	0	26.58333333	2			MSCNS	
634			bl	sep	Staphylococcus epidermidis	CoNS+	0	45.01666667	2			MSCNS	
635	m	1	bl	efm	Enterococcus faecium		1	24.68333333	2				
636			bl	sep	Staphylococcus epidermidis	CoNS+	0	81.68333334	4			MRCNS	
637			bl	sho	Staphylococcus hominis	CoNS++	0	38.78333333	2			MRCNS	
638			bl	sho	Staphylococcus hominis	CoNS+	0	30.06666666	2			MRCNS	

639	m	2	bl	efa	Enterococcus faecalis		1	24.06666667	2				
640	m	14	bl	sau	Staphylococcus aureus		1	14.96666667	1			MSSA	
641			bl	sep	Staphylococcus epidermidis	CoNS+	0	62.8	3			MRCNS	
642			bl	sho	Staphylococcus hominis	CoNS+	0	27.66666667	2			MRCNS	
643			bl	shl	Staphylococcus haemolyticus	CoNS+	0	43.11666666	2			MRCNS	
644			bl	sho	Staphylococcus hominis	CoNS++	0	24.31666667	2			MRCNS	
645			bl	sle	Staphylococcus sciuri	CoNS++	0	20.76666666	1			MRCNS	
646			bl	sho	Staphylococcus hominis	CoNS+	0	29.66666667	2			MSCNS	
647	f	2	bl	kpn	Klebsiella pneumoniae		1	9.233333333	1	+			
648			bl	sho	Staphylococcus hominis	CoNS+	0	43.18333333	2			MRCNS	
649	m	1	bl	efm	Enterococcus faecium		1	27.36666667	2				
650			bl	sep	Staphylococcus epidermidis	CoNS+	0	29.46666667	2			MSCNS	
651			bl	sep	Staphylococcus epidermidis	CoNS++	0	26.7	2			MRCNS	
652	m	7	bl	hin	Haemophilus influenzae		1	18.1	1				
653			bl	sep	Staphylococcus epidermidis	CoNS+	0	48.88333333	3			MSCNS	
654			bl	sep	Staphylococcus epidermidis	CoNS+	0	72.76666667	4			MRCNS	
655	m	1	bl	sep	Staphylococcus epidermidis	CoNS+	1	14.6	1			MRCNS	
656	m	2	bl	eco	Escherichia coli		1	10.8	1	+			
657	m	1	bl	sau	Staphylococcus aureus		1	12.3	1			MRSA	
658			bl	sho	Staphylococcus hominis	CoNS+	0	50.28333333	3			MSCNS	
659			bl	sho	Staphylococcus hominis	CoNS+	0	23.28333333	1			MRCNS	
660	m	6	bl	pma	Stenotrophomonas maltophilia		1	12.01666667	1				
661	m	2	bl	sgc	Streptococcus agalactiae		1	13.13333333	1				
662	m	3	bl	spn	Streptococcus Pneumoniae		1	11.43333333	1				PSSP
663			bl	sca	Staphylococcus capitis	CoNS+	0	44.2	2			MRCNS	
664			bl	shl	Staphylococcus haemolyticus	CoNS+	0	33.96666667	2			MRCNS	
665	f	2	bl	eco	Escherichia coli		1	17.85	1	+			
666	f	14	bl	efm	Enterococcus faecium		1	23.48333333	1				
667			bl	sep	Staphylococcus epidermidis	CoNS+	0	64.23333333	3			MRCNS	
668	f	2	bl	sep	Staphylococcus epidermidis	CoNS+	1	19.36666667	1			MRCNS	
669			bl	sho	Staphylococcus hominis	CoNS+	0	51.01666667	3			MRCNS	
670	f	2	bl	efa	Enterococcus faecalis		1	14.36666667	1				
671			bl	sho	Staphylococcus hominis	CoNS+	0	33.2	2			MRCNS	
672	m	7	bl	kpn	Klebsiella pneumoniae		1	7.716666667	1	+			
673			bl	shl	Staphylococcus haemolyticus	CoNS+	0	24.88333333	2			MRCNS	
674	f	3	bl	spn	Streptococcus Pneumoniae		1	12.73333333	1				PSSP
675	m	4	bl	kpn	Klebsiella pneumoniae		1	12.75	1	+	+		
676			bl	sca	Staphylococcus capitis	CoNS+	0	35.36666667	2			MSCNS	
677			bl	sep	Staphylococcus epidermidis	CoNS++	0	23.6	1			MRCNS	
678			bl	sho	Staphylococcus hominis	CoNS+	0	39.56666667	2			MRCNS	

679			bl	sho	Staphylococcus hominis	CoNS+	0	40.63333333	2			MRCNS	
680			bl	sho	Staphylococcus hominis	CoNS+	0	71.48333334	3			MRCNS	
681			bl	sep	Staphylococcus epidermidis	CoNS++	0	28	2			MSCNS	
682			bl	efm	Enterococcus faecium		0	44.93333333	2				
683			bl	pps	Staphylococcus saprophyticus	CoNS+	0	44.21666667	2			MRCNS	
684			bl	sep	Staphylococcus epidermidis	CoNS++	0	25.26666667	2			MSCNS	
685			bl	sep	Staphylococcus epidermidis	CoNS+	0	38.81666667	2			MRCNS	
686			bl	sep	Staphylococcus epidermidis	CoNS+	0	51.35	3			MRCNS	
687			bl	sep	Staphylococcus epidermidis	CoNS+	0	50.36666667	3			MRCNS	
688			bl	sho	Staphylococcus hominis	CoNS+	0	37.93333333	2			MRCNS	
689			bl	sho	Staphylococcus hominis	CoNS+	0	39.2	2			MRCNS	
690	m	2	bl	kpn	Klebsiella pneumoniae		1	15.33333333	1	-			
691			bl	spn	Streptococcus Pneumoniae		0	36.85	2				PSSP
692	m	7	bl	spn	Streptococcus Pneumoniae		1	28.63333333	2				PSSP
693			bl	efa	Enterococcus faecalis		0	34.75	2				
694	m	1	bl	sgc	Streptococcus agalactiae		1	4.283333333	1				
695	m	2	bl	kpn	Klebsiella pneumoniae		1	12.63333333	1	+			
696	m	2	bl	kpn	Klebsiella pneumoniae		1	9.199999999	1	+			
697	m	12	bl	pae	Pseudomonas aeruginosa		1	5.766666667	1		+		
698			bl	sau	Staphylococcus aureus		0	33.71666667	2			MSSA	
699	m	5	bl	sal	Salmonella		1	19.4	1				
700	m	1	bl	kpn	Klebsiella pneumoniae		1	9.3	1	-			
701			bl	sau	Staphylococcus aureus		0	34.68333333	2			MSSA	
702	m	2	bl	kpn	Klebsiella pneumoniae		1	37.65	2	-			
703	m	2	bl	kpn	Klebsiella pneumoniae		1	13.95	1	-			
704	f	1	bl	eco	Escherichia coli		1	9.866666667	1	-			
705	f	2	bl	pae	Pseudomonas aeruginosa		1	20.73333333	1				
706	f	3	bl	kpn	Klebsiella pneumoniae		1	10.63333333	1				
707	f	1	bl	sma	Serratia marcescens		1	13	1				
708	f	4	bl	sma	Serratia marcescens		1	17.7	1				
709	f	1	bl	kpn	Klebsiella pneumoniae		1	29.06666667	2				
710	f	10	bl	sau	Staphylococcus aureus		1	23.73333333	1			MSSA	
711	f	1	bl	sma	Serratia marcescens		1	68.63333333	3				
712	f	2	bl	kpn	Klebsiella pneumoniae		1	12.1	1				
713	f	1	bl	kpn	Klebsiella pneumoniae		1	19.05	1				
714	f	4	bl	spn	Streptococcus Pneumoniae		1	20.7	1				PISP
715	m	3	bl		Achromobacter xylosoxidans		1	16.26666667	1				
716	m	6	bl	sgc	Streptococcus agalactiae		1	7.05	1				
717	f	1	bl	sgc	Streptococcus agalactiae		1	17	1				
718	m	5	bl	sgc	Streptococcus agalactiae		1	20.75	1				

719	m	1	bl	spn	Streptococcus Pneumoniae		1	14.88333333	1				PSSP
720	f	4	bl	spn	Streptococcus Pneumoniae		1	10.43333333	1				PSSP
721	f	7	bl		Streptococcus sanguis		1	24.93333333	2				
722	f	1	bl	sgc	Streptococcus agalactiae		1	15.93333333	1				
723	f	9	bl	sgc	Streptococcus agalactiae		1	7.333333332	1				
724	m	5	bl	sgc	Streptococcus agalactiae		1	15.45	1				
725	m	13	bl	sgc	Streptococcus agalactiae		1	14.41666667	1				
726	f	12	bl	sgc	Streptococcus agalactiae		1	19.83333333	1				
727	m	3	bl	spn	Streptococcus Pneumoniae		1	16.05	1				PSSP
728	f	11	bl	pma	Stenotrophomonas maltophilia		1	14.85	1				
729	f	14	bl	sau	Staphylococcus aureus		1	14.21666667	1			MRSA	
730	f	13	bl	kpn	Klebsiella pneumoniae		1	8.266666666	1	+			
731			bl		Elizabethkingia menin-gosepticum		0	26.15	2				
732	f	12	bl	pma	Stenotrophomonas maltophilia		1	18.65	1				
733	m	1	bl	kpn	Klebsiella pneumoniae		1	13.83333333	1				
734	m	1	bl	aba	Acinetobacter baumannii		1	16.36666667	1	NON-XDR			
735	f	5	bl	efa	Enterococcus faecalis		1	16.96666667	1				
736	f	6	bl	kpn	Klebsiella pneumoniae		1	17.45	1	-			
737	m	4	bl	kpn	Klebsiella pneumoniae		1	24	1	+			
738	m	2	bl	kpn	Klebsiella pneumoniae		1	13.2	1	+			
739	f	1	bl	aba	Acinetobacter baumannii		1	40.23333333	2	NON-XDR			
740	m	2	bl	efm	Enterococcus faecium		1	24.51666667	2				
741	m	3	bl	sal	Salmonella		1	32.38333333	2				
742	m	4	bl	kpn	Klebsiella pneumoniae		1	13.28333333	1	-			
743	f	4	bl	pae	Pseudomonas aeruginosa		1	27.05	2				
744	m	5	bl	sal	Salmonella		1	12.91666667	1				
745	m	5	bl	sma	Serratia marcescens		1	12.1	1				
746	m	5	bl	sma	Serratia marcescens		1	16.58333333	1				
747	f	5	bl	aba	Acinetobacter baumannii		1	19.58333333	1	XDR			
748	m	3	bl	sal	Salmonella		1	9.233333333	1				
749	f	5	bl	efa	Enterococcus faecalis		1	14.8	1				
750	f	5	bl	sau	Staphylococcus aureus		1	19.03333333	1			MRSA	
751	m	1	bl	sau	Staphylococcus aureus		1	14.96666667	1			MRSA	
752	f	3	bl	hin	Haemophilus influenzae		1	14.93333333	1				
753	m	7	bl	kpn	Klebsiella pneumoniae		1	12.9	1	+			
754	m	2	bl	eco	Escherichia coli		1	11.48333333	1	+			
755	m	5	bl	pae	Pseudomonas aeruginosa		1	23.91666666	1				
756	f	5	bl	efa	Enterococcus faecalis		1	15.53333333	1				
757	m	1	bl	sau	Staphylococcus aureus		1	25.31666667	2			MSSA	
758			bl	sau	Staphylococcus aureus		0	34.88333334	2			MSSA	

759	f	5	bl	eco	Escherichia coli		1	16.43333334	1	+			
760	f	8	bl	aba	Acinetobacter baumannii		1	9.799999999	1	XDR			
761	f	1	bl	eco	Escherichia coli		1	15.55	1	+			
762	m	10	bl	kpn	Klebsiella pneumoniae		1	25.48333333	2	+			
763	m	1	bl	kpn	Klebsiella pneumoniae		1	14.88333333	1	-			
764	m	3	bl	sau	Staphylococcus aureus		1	36	2			MSSA	
765	f	2	bl	sau	Staphylococcus aureus		1	15.06666667	1			MRSA	
766			bl	efm	Enterococcus faecium		0	44.6	2				
767	f	1	bl	kpn	Klebsiella pneumoniae		1	22.35	1	+			
768	m	2	bl	eco	Escherichia coli		1	11.85	1	+			
769			bl		Bacillus cereus		0	24.95	2				
770			bl		Gram-positive bacilli		0	63.65	3				
771			bl		Bacillus cereus		0	14.75	1				
772			bl		Bacillus cereus		0	13.75	1				
773			bl	mlu	Micrococcus luteus		0	44.45	2				
774			bl	mlu	Micrococcus luteus		0	33.63333333	2				
775			bl		Bacillus		0	21.68333333	1				
776			bl		Gram-positive bacilli		0	53.96666667	3				
777			bl		Bacillus cereus		0	12.76666667	1				
778			bl		Bacillus cereus		0	22.81666667	1				
779			bl		Bacillus		0	28.58333333	2				
780			bl		Gram-positive bacilli		0	28.15	2				
781			bl		Bacillus cereus		0	18.2	1				
782	m	13	bl	cal	Candida albicans		1	34.33333333	2				
783	m	13	bl	cal	Candida albicans		1	33.13333333	2				
784	m	3	bl	cal	Candida albicans		1	14.1	1				
785	f	7	bl		Candida parapsilosis		1	53.96666667	3				
786	f	1	bl		Candida parapsilosis		1	82.25	4				
787	m	7	bl		Candida parapsilosis		1	46.56666667	2				
788	f	7	bl	cal	Candida albicans		1	62.4	3				
789	m	2	bl	cal	Candida albicans		1	31.31666667	2				
790	m	2	bl	cal	Candida albicans		1	30.63333333	2				
791	m	1	bl		Candida parapsilosis		1	26.53333333	2				
792	f	1	bl		Candida parapsilosis		1	24.55	2				
793	m	2	bl	cal	Candida albicans		1	33.75	2				
794	m	2	bl	cal	Candida albicans		1	35.75	2				
795	f	2	bl	cal	Candida albicans		1	34.86666667	2				
796	f	2	bl	cal	Candida albicans		1	32.03333333	2				
797	f	4	bl		Candida glabrata		1	31.13333334	2				
798	f	13	bl		Candida parapsilosis		1	16.88333333	1				

799	m	13	bl		Candida glabrata		1	67.21666667	3				
800	m	1	bl		Candida parapsilosis		1	32.8	2				
801	m	1	bl		Candida parapsilosis		1	30.13333333	2				
802	f	1	bl		Candida parapsilosis		1	32.78333333	2				
803	m	4	bl		Candida parapsilosis		1	34.28333333	2				
804	m	5	bl	cal	Candida albicans		1	27.33333333	2				
805	f	7	bl		Candida parapsilosis		1	30.7	2				
806	f	9	bl		Candida parapsilosis		1	24.7	2				
807	f	2	bl	cal	Candida albicans		1	27.65	2				
808	m	1	bl		Candida parapsilosis		1	31.46666667	2				
